# The influence of external electric fields on proton transfer tautomerism in the guanine–cytosine base pair†

**DOI:** 10.1039/d0cp06218a

**Published:** 2021-02-23

**Authors:** Alexander Gheorghiu, Peter V. Coveney, Alya A. Arabi

**Affiliations:** Centre for Computational Science, University College London 20 Gordon St London WC1H 0AJ UK p.v.coveney@ucl.ac.uk +44 20 7679 5300; Informatics Institute, University of Amsterdam P.O. Box 94323 1090 GH Amsterdam The Netherlands; College of Medicine and Health Sciences, Biochemistry Department, United Arab Emirates University AlAin P. O. Box: 17666 United Arab Emirates alya.arabi@dal.ca

## Abstract

The Watson–Crick base pair proton transfer tautomers would be widely considered as a source of spontaneous mutations in DNA replication if not for their short lifetimes and thermodynamic instability. This work investigates the effects external electric fields have on the stability of the guanine–cytosine proton transfer tautomers within a realistic strand of aqueous DNA using a combination of ensemble-based classical molecular dynamics (MD) coupled to quantum mechanics/molecular mechanics (QM/MM). Performing an ensemble of calculations accounts for the stochastic aspects of the simulations while allowing for easier identification of systematic errors. The methodology applied in this work has previously been shown to estimate base pair proton transfer rate coefficients that are in good agreement with recent experimental data. A range of electric fields in the order of 10^4^ to 10^9^ V m^−1^ is investigated based on their real-life medicinal applications which include gene therapy and cancer treatments. The MD trajectories confirm that electric fields up to 1.00 × 10^9^ V m^−1^ have a negligible influence on the structure of the base pairs within DNA. The QM/MM results show that the application of large external electric fields (1.00 × 10^9^ V m^−1^) parallel to the hydrogen bonds increases the thermodynamic population of the tautomers by up to one order of magnitude; moreover, the lifetimes of the tautomers remain insignificant when compared to the timescale of DNA replication.

## Abstract 2

A detailed investigation is reported of mutagenic guanine–cytosine tautomerization in a realistic model of DNA bathed in intense electric fields using a combination of classical molecular dynamics and quantum mechanics/molecular mechanics methods.

## Graphical abstract

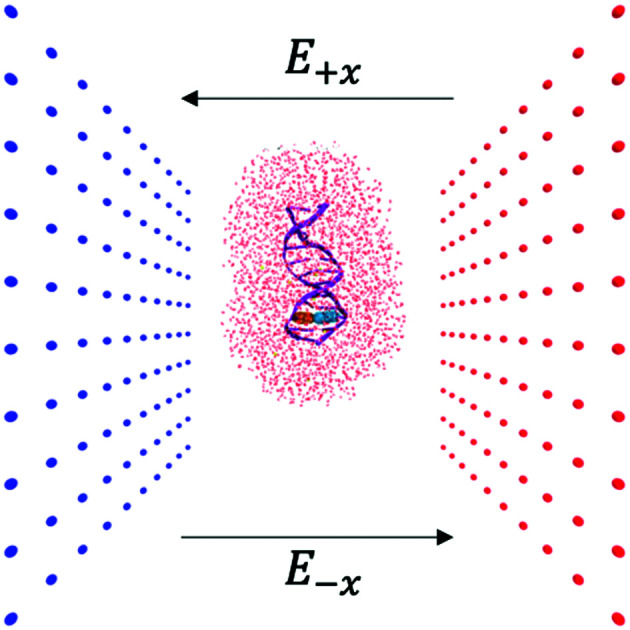

## 1 Introduction

**Fig. 1:**
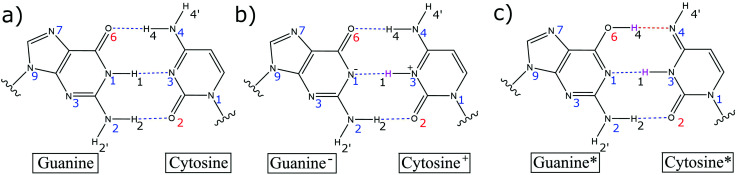
(a) Canonical Watson–Crick GC base pair, (b) the single proton transfer zwitterion tautomer G^−^C^+^ and (c) the double proton transfer G*C* tautomer (imino-enol). Transferred hydrogen atoms are highlighted in pink.

It was Löwdin who proposed that the Watson–Crick base pairs (GC and AT) proton transfer imino-enol tautomers (G*C* and A*T*) facilitate base pair mismatches (CA*, C*A, GT*, G*T) during the DNA replication process (Fig. 1).^1^ These tautomeric mismatches, provided they remain undetected by the various repair mechanisms, are thought to be a source of single point mutations, *i.e.*, GC → AT mutations, during the replication process. This hypothesis is supported by X-ray crystallography structures,^2^ which show that the tautomeric C*A mismatch within a DNA duplex in the insertion site of DNA polymerase has a similar geometry to the canonical base pairs and is therefore, a likely source of mutations. The biggest impediment to the Löwdin mechanism of mutation lies within the stability of the initial proton transfer imino-enol tautomers (G*C* and A*T*). Recently, NMR spectroscopy has provided an estimate for the tautomeric rate coefficients for certain base pair mismatches (G*T and G*U) in aqueous nucleic acids.^3,4^ Nonetheless, there is still a lack of experimental evidence that is specific to the canonical Watson–Crick base pair tautomerism.

The proton transfer tautomers are too short-lived to be thoroughly investigated by standard experimental techniques and for this reason, researchers have turned to theoretical techniques. Recent computational studies have calculated the kinetic and thermodynamic properties of the Watson–Crick base pair tautomers to be highly unstable using density functional theory (DFT)^5^ and DFT quantum mechanical/molecular mechanical (QM/MM) techniques.^6^ The simulations predict the G*C* tautomer to have a very small concentration at equilibrium (*K* = 10^−9^) and the fast kinetics of the reverse reaction (G*C* → GC) promote the swift reverting of the tautomer to canonical GC. In addition, owing to the extremely short lifetime of G*C*, *i.e.*, in the range of femtoseconds to picoseconds,^6^ the lifetime of the transient tautomer is approximately three to five orders of magnitude smaller than the nanoseconds it takes for DNA to unwind during the replication process.^7^ As such, the contribution of base pair tautomerism towards the rates of spontaneous mutations in DNA is considered to be negligible at best.

Researchers have used DFT and QM/MM to investigate how different external factors, *e.g.*, intercalation with *cis*-platin,^8^ analogous nucleobases,^9^ and intense external electric fields^10–12^ affect the stability of the proton transfer tautomers. In addition, a wide range of studies have investigated how the population of different excited states influence the mechanism of proton transfer in the absence^13–15^ and presence of external electric fields.^16,17^ The effects of excited state chemistry are beyond the scope of this study and warrant a future investigation. This work will primarily focus on the effects of external electric fields on base pair tautomerism at strengths that correspond to those found in real-life applications and scenarios. Electric fields possess therapeutic medical properties, some of which include the treatment of cancer,^18,19^ enhanced wound healing,^20,21^ and gene therapy.^22–24^ Modern electrochemotherapy approaches utilise electric fields of strengths 10^6^–10^7^ V m^−1^ in nanosecond pulses^25,26^ to temporarily permeabilise the tumour cell membrane for the targeted delivery of non-permeable drugs.^23^ Larger electric field strengths (10^8^–10^9^ V m^−1^)^27,28^ are naturally generated by the alignment of dipolar lipid residues with water molecules within fully saturated phospholipid membranes. More intense electric fields, larger than 10^9^ V m^−1^, are generated from the tip of a scanning tunnelling microscope (STM) during the imaging process.^29,30^ Experimental STM data, in conjunction with theoretical studies, show that electric fields of the order 10^9^ V m^−1^ and above are large enough to cause water molecules to align by their dipoles, rather than the distinctive hydrogen-bonded network.^30^

Previous theoretical studies have shown that large oriented external electric fields (*ca.* 2 × 10^9^ V m^−1^ or larger) aligned parallel to the base pair hydrogen bonds, drastically influence the kinetics and thermodynamics of the base pair proton transfer reactions.^10–12^ For example, Arabi and Matta demonstrated that an electric field of 2.54 × 10^9^ V m^−1^ dynamically stabilised the G*C* tautomer, while Cerón-Carrasco and Jacquemin^11^ showed that the single proton transfer G^−^C^+^ zwitterion is instead stabilised. These calculations have led to the conclusion that in the presence of specifically oriented large external electric fields, base pair tautomerism may be considered as a viable mutation mechanism. However, the current models available in the literature are limited by the study of base pairs in the gas phase and do not account for a realistic biological environment. Shaik *et al.*^16^ recently reviewed the prospects of external electric fields as future smart reagents in chemistry and concluded that the accurate modelling of solvent effects requires a combination of both molecular dynamics simulations and quantum mechanical methods. Indeed, a gas phase study does not account for the important interactions that occur between water molecules in the presence of large electric fields.^30^ Previous gas-phase models also report their findings without any error quantification, the importance of which is paramount as the data required to validate the simulation results against those obtained in experimental methods is sparse.

In this paper, we will utilise all-atom molecular dynamics to determine the effect of external electric fields on the structural properties of a realistic aqueous DNA system. In addition, we will use QM/MM to investigate the influence external electric fields have on the rate of single point mutations in aqueous DNA *via* the proton transfer imino-enol tautomerism in the Watson–Crick GC base pair. We have shown in our previous work^6^ that this combination of multiscale techniques yields activation energy barriers and rate coefficients for GC proton transfer that are in better agreement with recent NMR experiments^3,4^ than alternative gas-phase QM models. The errors in our simulations are quantified by performing multiple replicas *via* the application of an ensemble-based methodology.

This paper will address three different base pair tautomerism pathways that occur in the GC base pair:

(1) Concerted double proton transfer1
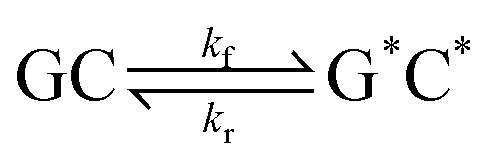
to form the double proton transfer tautomer G*C*; the forward and reverse rate coefficients are given by *k*_f_ and *k*_r_, respectively.

(2) Stepwise double proton transfer2
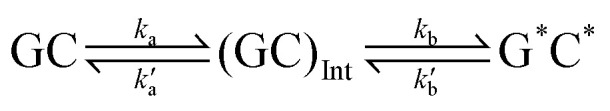
a two-step mechanism which proceeds *via* a single proton transfer intermediate (GC)_Int_. The rate coefficients pertaining to the first and second steps are embellished by the subscripts ‘a’ and ‘b’, respectively.

(3) Concerted single proton transfer3
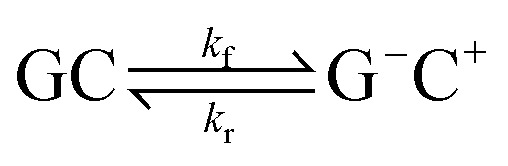
whereby G^−^C^+^ is the zwitterionic product.

## 2 Materials and methods

### 2.1 Equilibrating the system

A crystal structure of a double-stranded B-DNA dodecamer (PDB ID: 1BNA) was used to construct the simulating systems for this work. Using the AmberTools package, the system was neutralised with 22 sodium ions (Na^+^) and fully solvated in a box (dimensions: 71.15 Å × 73.13 Å × 85.94 Å) using the TIP3P water model.^31^ Ensemble-based all atom molecular dynamics (MD) simulations were then performed under periodic boundary conditions using the NAMD code.^32^ The energies of the system were calculated using the AMBER parmbsc1 force field,^33^ as it has been shown to accurately describe the long timescale dynamics (up to tens of μs) for solvated DNA systems.^34,35^ The cutoff interaction distance for the van der Waals and the electrostatics were set to 10.0 Å excluding pairs of atoms further than 11.5 Å apart. At distances beyond the cutoff, the electrostatics were calculated using the particle mesh Ewald method with a grid spacing set to 1 Å. The following equilibration protocol was then performed: First, the coordinates of the DNA double helix were restrained, whilst the geometry of the rest of the system was minimised using the conjugate gradient and line search algorithm. The temperature of the system was then gradually increased from 50 K to 300 K over the course of 30 ps using a time step of 1 fs. The temperature of the system was maintained at 300 K using a Berendsen barostat at a pressure of 1 atm. The restraints on the DNA were then gradually removed over 0.5 ns, followed by an unrestrained 0.5 ns run. Once the system was equilibrated, a fully unrestrained 10 ns production run was performed at constant pressure (1 atm) and temperature (300 K) with a time step of 2 fs. The bonds between heavy atoms and hydrogen atoms were constrained to their nominal length using the SHAKE algorithm. The equilibration and production runs were individually repeated ten times to form an ensemble of ten replicas and a total of 100 ns simulation.

During the production runs, a constant external electric field was then applied in a specific direction. We individually assessed six different electric field directions along the principle simulation cell axis (*E*_+*x*_, *E*_−*x*_, *E*_+*y*_, *E*_−*y*_, *E*_+*z*_ and *E*_−*z*_) at different strengths (increasing by an order of magnitude from 1.00 × 10^5^ V m^−1^ to 1.00 × 10^9^ V m^−1^). Using the protocol as described above an ensemble of MD simulations were performed with the inclusion of an electric field. A total of 300 independent 10 ns simulation trajectories (six electric field directions, five electric field strengths and ten replicas of each) were obtained. The MD simulations in the absence of the electric field, which are used as a comparison to those performed in this work, were obtained in our previous study^6^ using an identical methodology. The MD simulations in the presence of the electric fields were carried out on the Dutch national supercomputer Cartesius using NAMD 2.12.

### 2.2 Calculating the proton transfer kinetics

The QM/MM simulations were performed using the ChemShell^36^ package to couple the QM and density functional theory (DFT) code NWChem^37^ with the MM code DL-POLY.^38^ All of the QM/MM routines were performed using the DL-FIND module^39^ as implemented in ChemShell. Initial configurations for the QM/MM simulations were drawn from the combined classical MD trajectories in the absence of the electric field. A total of 25 different configurations were chosen on a distance-based criterion between the nucleobases in the base pair of interest. As shown in our previous work,^6^ an ensemble of 25 QM/MM replicas is more than enough to achieve a suitable convergence in base pair proton transfer energies, with associated errors as low as 0.25 kcal mol^−1^.

The periodicity of each configuration was then removed so that only a solvation sphere of 15 Å around the DNA remained. Das *et al.* have shown that increasing the size of the QM region has a relatively small influence (*ca.* 1 kcal mol^−1^) on the energetic profile of base pair proton transfer.^40^ On the other hand, we have previously shown that the mean base pair proton transfer energy calculated from a sample of 25 QM/MM replicas can have an associated standard deviation of up to *ca.* 2.5 kcal mol^−1^.^6^ In other words, the accuracy that is gained from using a larger QM region is lost within the uncertainty of the proton transfer itself. For this reason, the QM subsystem consisted of a single GC base pair (residue number 3 and 22) with hydrogen linker-atoms placed between the deoxyribose carbon (C1′) and the corresponding terminal nitrogen of the nucleobase. Thus, the MM subsystem consisted of everything else, including the remaining DNA helix, the bulk solvent, and the sodium counter ions. The energy of the MM region was calculated using the AMBER parmbsc1 force field. The electrostatic coupling between the two subsystems was approximated using the electrostatic embedding technique so that the charges in the MM region polarise the QM electron density. The energy of the QM region was calculated using the hybrid exchange–correlation functional, B3LYP,^41^ and the Dunning aug-cc-pvdz basis set^42^ in combination with the exchange-hole dipole moment dispersion (XDM) model.^43^ In our previous study,^6^ we have shown that the B3LYP+XDM/aug-cc-pvdz QM method provides an adequate description of base pair geometries and calculates the dispersion interactions with remarkable accuracy when compared to gold-standard coupled-cluster reference values.^44^ A more thorough benchmark performed by Otero-de-la-Roza and Johnson^45^ also concludes that B3LYP is the hybrid functional of choice for studying reactions in organic molecules.

During geometry optimisation procedures, all residues within 15 Å of the QM region were free to move and the remaining residues were frozen in space. First, the reactant (GC) was optimised at the QM/MM level. The H4 and H1 protons (which correspond to the respective O6–H4–N4 and N1–H1–N3 hydrogen bond bridges) were then simultaneously transferred to the adjacent base to generate initial estimates for the geometry of the product (G*C*). From there, the same QM/MM protocol was employed to optimise the geometry of the DPT G*C* tautomer. In some cases, the geometry optimisation algorithm failed to locate a minimum that corresponded to G*C*; instead, the outer proton (H4) returned back towards the cytosine, which resulted in convergence of the geometry to the zwitterion SPT product (G^−^C^+^). The energetics of the proton transfer pathways were calculated using the climbing image nudged elastic band (CI-NEB)^46^ technique between the QM/MM optimised canonical base pair and proton transfer product. The reaction was then categorised as either a stepwise or concerted double proton transfer (DPT) pathway, or a single proton transfer (SPT) pathway, depending on the profile of the reaction coordinate and the geometry of the product. To calculate the thermodynamics and kinetics of the process, the transition states were further optimised using the dimer method^47^ and verified by a single imaginary frequency in the Hessian. For the stepwise processes, the geometry of the intermediate was also optimised to its local minimum. The Hessian for each optimised structure along the reaction pathway was then calculated using thermal corrections at 300 K. The harmonic approximation is a widely used and efficient method of approximating the Gibbs energy of a system.^5,6,10,12^ We note that using a sampling method is expected to reduce the source of errors within the approximation to the Gibbs energy. The proton transfer equilibrium constant is calculated using the following equation:

4 *K* = e^−Δ*G*/*RT*^

where Δ*G* is the Gibbs energy of reaction, *R* is the gas constant, and *T* is the temperature (300 K). The rate module in ChemShell was then used to calculate the rate coefficients for the proton transfer reactions using harmonic transition state theory (TST)^48^ and the tunnelling corrections were approximated using the Wigner correction at second order.^49^ The harmonic TST approximation should not be used to estimate the rate coefficients for processes with negative barriers. For this reason, we calculate the average tautomer half-life and proton transfer rate coefficients using only the QM/MM replicas that have a positive reverse Gibbs energy barrier. We encourage future studies to consider the use of the variable-reaction-coordinate variational TST (VRC-VTST)^50^ method since it provides an accurate description for the kinetics of processes without a barrier.^51^

**Fig. 2:**
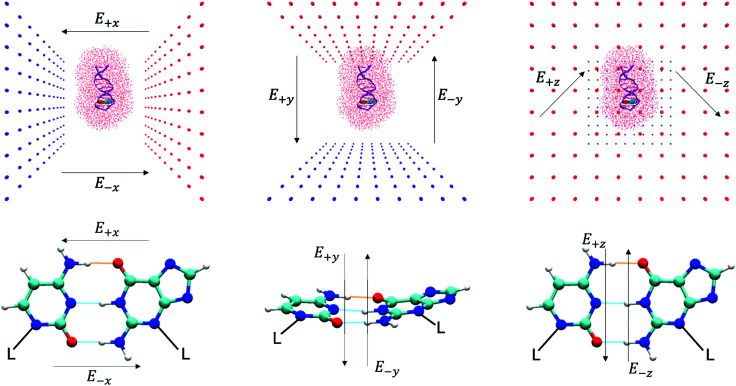
The external electric field directions (from left to right: *E*_+*x*_, *E*_−*x*_, *E*_+*y*_, *E*_−*y*_, *E*_+*z*_, and *E*_−*z*_) with respect to the entire DNA duplex in the QM/MM simulation (top), and the GC base pair (residues 3 and 22) in the QM region (bottom). The top row shows a typical QM/MM replica: the DNA dodecamer (purple cartoon representation), the GC base pair at the QM level (CPK representation, cytosine in orange and guanine in teal), the surrounding solvent and ions (red line representation) and the surrounding dummy atom point charges that apply the external electric field (red and blue balls). The GC base pair hydrogen bonds are aligned to the *xz*-axis and centred at the origin.

An oriented external electric field was then applied in six directions across the GC base pair, *E*_+*x*_, *E*_−*x*_, *E*_+*y*_, *E*_−*y*_, *E*_+*z*_ and *E*_−*z*_ (see Fig. 2 for reference) in different strengths, ranging from 1.00 × 10^4^ to 1.00 × 10^9^ V m^−1^. Each electric field direction corresponds to an independent ensemble of 25 QM/MM replicas, numerically labelled from 101 to 125. The field-free (*E*_0_) QM/MM results that are used as a comparison in this paper are the same as those that have been obtained in our previous work using an identical methodology.^6^ All QM/MM calculations were performed using ChemShell 3.7 and NWChem 6.6 on the UK national supercomputer ARCHER, and the UCL high-performance computing (HPC) facility Kathleen.

### 2.3 Electric field nomenclature

The ChemShell package lacks a distinct implementation of an integrated external electric fields module. Therefore, the external electric fields in QM/MM simulations in this work are generated by two oppositely charged plates that are positioned 100 Å apart to enclose the entire system. Each plate consisted of 100 point charges which are represented by non-interacting dummy atoms and are uniformly spaced in a 100 Å × 100 Å grid. The charges on each set of dummy atoms were systematically increased by order of magnitude (from 1.82 × 10^−6^ a.u. to 0.182 a.u.) to simulate different field strengths (1.00 × 10^4^ V m^−1^ to 1.00 × 10^9^ V m^−1^). Details of how the strength and homogeneity of the electric field were quantified are given in Table S1 and Fig. S1 of the ESI.† The base pair hydrogen bonds of interest are centred at the cell origin and aligned along the principal *xz*-axis to ensure that the external electric field strengths are consistent between QM/MM replicas. The charged plates are then oriented on different planes to generate different electric field directions (as shown in Fig. 2).

## 3 Results

### 3.1 Structure of DNA within external electric fields

**Fig. 3:**
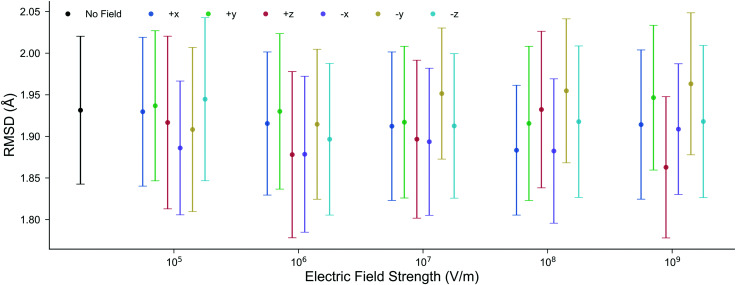
The mean RMSD for all non-hydrogen nucleic atoms compared to the X-ray crystal structure (1BNA from the Protein Data Bank^52^) at various electric field strengths and directions. Each point is the average RMSD of ten, 10 ns, all-atom MD replicas; the error bars represent the standard deviation. The mean RMSD in the presence of the electric fields is well within the boundary of error associated with the RMSD in the absence of the electric field.

The structure of DNA during the MD simulations is quantified by two measurements: (i) the root-mean-squared-deviation (RMSD) of all non-hydrogen atoms compared to the X-ray structure,^52^ and (ii) the average lengths of the inter-base pair hydrogen bonds. In ambient conditions, a low mean RMSD (≤2 Å) for an MD trajectory ensures that the entire DNA structure has correctly equilibrated. It is shown in Fig. 3 that the mean RMSD of the DNA structure in all of the electric field strengths and directions are well within the standard deviation error of the RMSD in the absence of the field. Changing the electric field direction, *e.g.*, moving from *E*_+*x*_ to *E*_−*x*_, or *E*_−*y*_ does not correlate with the RMSD of the DNA structure. We also find that the average GC hydrogen bond lengths in the presence of the external electric fields differ by less than 0.015 Å to those in the absence (further details are given in Fig. S2 of the ESI†). Overall, there are no noticeable differences in the structure of DNA during the 10 ns classical MD simulations in the absence, or the presence, of electric fields up to 1.00 × 10^9^ V m^−1^. For this reason, the subsequent QM/MM simulations will use geometries taken from the MD trajectories in the absence of the electric fields.

### 3.2 Proton transfer in weak external electric fields

Two ‘weak’ electric field strengths are chosen based on their practical medicinal applications (1.00 × 10^4^ V m^−1^ and 1.00 × 10^7^ V m^−1^),^53–58^ to ensure that they safely interact with the DNA of a patient. As shown in Table 1, these weaker oriented external electric fields (1.00 × 10^4^ V m^−1^ and 1.00 × 10^7^ V m^−1^) were found to have a negligible influence on the energetics of proton transfer in the GC base pair. As such, the distribution of proton transfer reactions occurring within the GC base pair (given in Table 2) remained equal to those in the absence of the external electric field (*E*_0_). The CI-NEB reaction coordinates for each QM/MM replica (provided in Fig. S3 of the ESI†) show that the field-free and the weak external electric field reaction coordinates are almost indistinguishable from one another.

**Table 1:** The electronic energies of stepwise double proton transfer (DPT), concerted DPT, and single proton transfer (SPT) in GC calculated using the B3LYP+XDM/aug-cc-pvdz/AMBER QM/MM method. The electronic energy for the first and second transition states (TS1 and TS2) and the reaction energy for each process are given in kcal mol^−1^ relative to the canonical GC base pair. The mean energies are calculated from the QM/MM ensemble and *σ* is the standard deviation (where *σ* = ‘—’, the sample size consists of a single replica). The electric field strengths are given in V m^−1^ and applied in the +*x*-direction. In the case of no field, as denoted by ‘0’, are those reported in earlier work^6^ and were calculated using the same QM/MM method

Electric field (V m^−1^)	Stepwise DPT						Concerted DPT				SPT			
	TS1		TS2		Reaction		TS1		Reaction		TS1		Reaction	
	Mean	*σ*	Mean	*σ*	Mean	*σ*	Mean	*σ*	Mean	*σ*	Mean	*σ*	Mean	*σ*
0.00	14.26	1.19	15.33	1.29	13.76	1.11	16.26	2.13	12.06	0.58	11.47	—	9.18	—
1.00 × 10^4^	14.33	1.22	15.40	1.31	13.76	1.11	16.45	2.38	12.05	0.59	11.45	—	9.17	—
1.00 × 10^7^	14.12	1.19	15.21	1.42	13.39	1.92	16.34	2.08	12.05	0.59	11.26	—	9.13	—

**Table 2:** The ratio of proton transfer reaction pathways observed in a GC base pair under the influence of an electric field applied along the three principal axes of the hydrogen bonds at 1.00 × 10^9^ V m^−1^. The orientations for each OEEF direction are shown in Fig. 2. The ratio of reaction pathways is based on the CI-NEB reaction coordinates for 25 QM/MM replicas per electric field direction

Electric field direction	Single proton transfer	Double proton transfer		
		Total	Stepwise	Concerted
*E*_−*x*_	0	25	8	17
*E*_0_ a	1	24	21	3
*E*_+*x*_	14	11	10	1

a Electric fields in the positive and negative *y*- and *z*-directions (*E*_+*y*_, *E*_−*y*_, *E*_+*z*_, and *E*_−*z*_) as well as the weaker electric fields (1.00 × 10^4^ V m^−1^ and 1.00 × 10^7^ V m^−1^) exhibit the same ratio of proton transfer reactions as in the *E*_0_ case.

Therefore, we confirm that mutations *via* base pair proton transfer do not occur more readily in the context of therapeutic medical treatments, as these rarely exceed 10^7^ V m^−1^. The results presented here reinforce the predictions made from previous computational studies, many of which have shown that external electric fields ∼5 × 10^8^ V m^−1^ do not influence the energetics of proton transfer reactions.^10–12,59–62^

### 3.3 Proton transfer in strong external electric fields

As given in Fig. 2, there are three main axes about the GC base pair hydrogen bonds of which a strong external electric field (1.00 × 10^9^ V m^−1^) was applied. The distribution of proton transfer reactions in the presence of different oriented external electric fields at 1.00 × 10^9^ V m^−1^ is given in Table 2. The most notable effects occur when the electric field is along the *x*-direction, *i.e.*, the direction parallel to the hydrogen bond axis (*E*_+*x*_ and *E*_−*x*_). The external electric fields oriented orthogonal to the hydrogen bond axis (*E*_+*y*_, *E*_−*y*_, *E*_+*z*_, and *E*_−*z*_) reported a ratio of proton transfer reactions that were identical to those observed in the field-free scenario (*E*_0_). Overall, the orthogonal electric fields were found to have a very small influence on the energetics of the GC proton transfer tautomerism. The majority of reaction coordinates in the *E*_±*y*_ and *E*_±*z*_ electric fields were similar to the field-free case (the corresponding CI-NEB reaction pathways are given in Fig. S4 and S5 of the ESI,† respectively). Since the thermodynamics and kinetics of the proton transfer process in both the *E*_±*y*_ and *E*_±*z*_ fields at 1.00 × 10^9^ V m^−1^ are presumed to be similar to the field-free (*E*_0_) instance, their transition state estimates were not subjected to further geometry optimisation. The finding that external electric fields orthogonal to the hydrogen bond axis have a negligible effect on proton transfer reaction energies is consistent with previous gas phase QM simulations.^12,61–63^

### 3.4 Electric fields parallel to the hydrogen bonds

The ratio of proton transfer reactions in GC (given in Table 2) substantially differs between the instances of *E*_+*x*_, *E*_−*x*_, and *E*_0_. The *E*_+*x*_ field favours the formation of the single proton transfer zwitterion G^−^C^+^ and occurs roughly 40% more frequently than the stepwise double proton transfer G*C* tautomer. The most rarely observed proton transfer pathway in the *E*_+*x*_ field is the concerted process, which occurred only once. By contrast, the *E*_−*x*_ field strongly favours the formation of the neutral G*C* tautomer over the G^−^C^+^ zwitterion; there were zero scenarios of single proton transfer altogether. When compared to the *E*_0_ scenario, the *E*_−*x*_ concerted double proton transfer mechanism occurred roughly six times more frequently, with stepwise double proton transfer being the subsidiary mechanism.

**Fig. 4:**
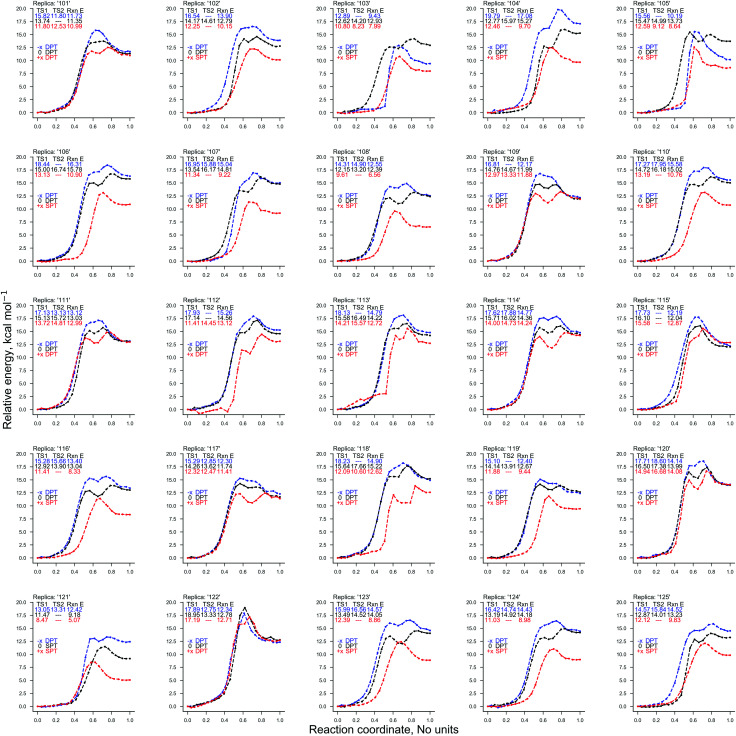
The electronic energy of the 25 QM/MM CI-NEB reaction coordinates for the GC proton transfer reactions: in the absence of external electric fields (black) and the presence of an external electric field (1.00 × 10^9^ V m^−1^) in the *E*_+*x*_ (red) and *E*_−*x*_ (blue) directions. The reaction coordinate is normalised (no units) and the electronic energy relative to the reactant (GC) is given in kcal mol^−1^.

The electric fields applied parallel to the GC base pair hydrogen bonds (*E*_+*x*_ and *E*_−*x*_), were found to have opposite effects on the energetics of the proton transfer tautomerism reactions depending on the polarity of the field. There is a trend whereby *E*_−*x*_ increases and *E*_+*x*_ decreases the relative electronic proton transfer reaction energy (Δ*E*) when compared to *E*_0_. The CI-NEB reaction coordinates given in Fig. 4 demonstrate that for more than half the replicas, the *E*_+*x*_ field reduces Δ*E* by *ca.* 5 kcal mol^−1^. This is due to the *E*_+*x*_ field making single proton transfer reactions occur more frequently than double proton transfer. This large charge in CI-NEB reaction coordinate energetics suggests that the *E*_+*x*_ and *E*_−*x*_ fields may largely influence the kinetics and thermodynamics of the proton transfer reactions when compared to the *E*_0_ instance. For this reason, the approximate transition states in the *E*_+*x*_ and *E*_−*x*_ fields were further optimised using the dimer method, and the new rate coefficients were calculated.

**Fig. 5:**
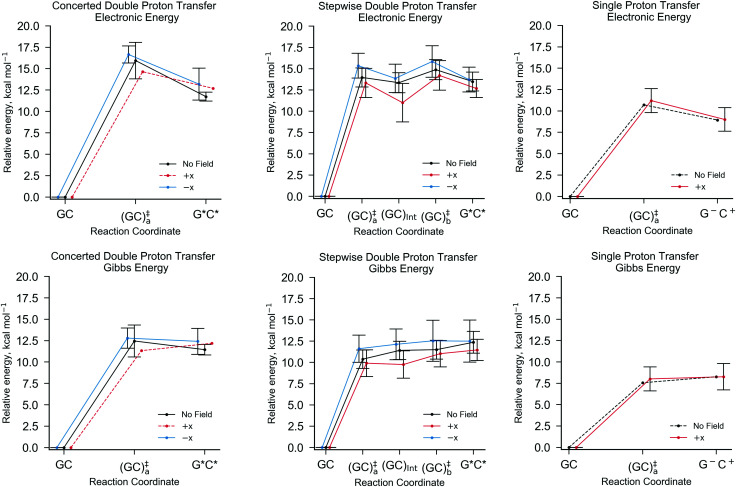
The QM/MM (B3LYP+XDM/aug-cc-pvdz/AMBER) optimised reaction coordinates for different GC proton transfer reactions in the absence of external electric fields (black), and the presence of an external electric field (1.00 × 10^9^ V m^−1^) in the positive (red) and negative (blue) *x*-directions. From left to right, concerted double proton transfer, stepwise double proton transfer, and single proton transfer. The top row shows the electronic energies and the bottom row the Gibbs energies relative to the canonical GC. The error bars are the standard deviation of the mean values. In the case of no reported errors, as demonstrated by dashed lines, no standard deviation was calculated due to only one replica of the QM/MM-ensemble exhibiting such a reaction pathway.

Fig. 5 shows the relative energies of each QM/MM geometry optimised stationary point in the three different types of proton transfer reactions that occur within the GC base pair. The Gibbs energy correction to the electronic energy lowers the relative energy of the transition states by *ca.* 2.5 kcal mol^−1^, whilst the reaction energy is comparatively reduced by no more than 0.5 kcal mol^−1^. The average Gibbs energy of the proton transfer products (and intermediate) are therefore higher in energy than the transition states; in the majority of proton transfer scenarios, the reverse Gibbs energy barrier is either very small, or negative and thus, a ‘barrierless’ process. Nevertheless, the standard deviation errors associated with the mean Gibbs energy barriers (*ca.* ±1 kcal mol^−1^) are large enough to suggest that in some cases, the reverse barrier may be greater than zero. As mentioned before, the tautomer half-life and rate coefficients reported in this paper using harmonic TST are calculated using only the QM/MM replicas with positive reverse Gibbs energy barriers.

#### 3.4.1 Stepwise double proton transfer

**Fig. 6:**
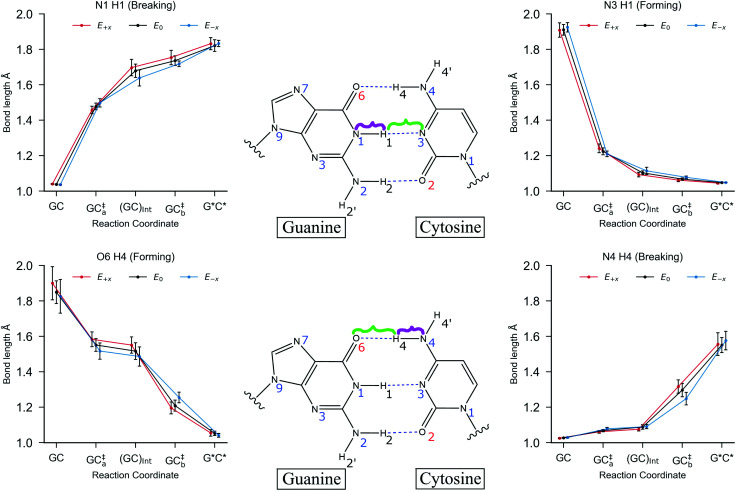
The mean hydrogen bond lengths for each stationary point in the stepwise double proton transfer reaction in GC. The geometries for each stationary point are optimised using B3LYP+XDM/aug-cc-pvdz/AMBER. The bonds being broken (N4–H4 and N1–H1) are highlighted in purple, and the bonds that are formed (O6–H4 and N3–H1) are highlighted in green. The external electric fields (*E*_+*x*_ in red and *E*_−*x*_ in blue) are applied at 1.00 × 10^9^ V m^−1^, and no field (*E*_0_) is given in black. The error bars are the standard deviation of the mean bond lengths.

The transfer of protons during the stepwise double proton transfer process is asynchronous, that is, one proton is transferred and sequentially followed by the transfer of another. This asynchronous nature is demonstrated in Fig. 6, whereby the N1–H1 bond breaks first (towards the formation of the intermediate, GC_Int_), followed by the breaking of the N4–H4 bond (to form tautomer product, G*C*). The *E*_+*x*_ and *E*_−*x*_ electric fields have a negligible impact on the initial GC N4–H4 and N1–H1 bonds when compared to *E*_0_.

Table 3 details the thermodynamics and kinetics of the stepwise process in the presence of *x*-direction electric fields at 1.00 × 10^9^ V m^−1^. Generally speaking, the *E*_+*x*_ field affects the energetics of the stepwise double proton transfer reaction in an equal and opposite way to the *E*_−*x*_ field. More specifically, the relative electronic and Gibbs energy of each stationary point on the stepwise double proton transfer reaction coordinate (GC_a_, GC_Int_, GC_b_ and G*C*) relative to the reactant (GC) follow a trend, whereby *E*_−*x*_ > *E*_0_ > *E*_+*x*_. An example of this trend is given in Table 3: the mean relative Gibbs energy of the first transition state (GC^‡^_a,f_) is 11.61 kcal mol^−1^ at *E*_+*x*_, 10.38 kcal mol^−1^ at *E*_0_, and 9.91 kcal mol^−1^ at *E*_−*x*_. The optimised reaction coordinate in Fig. 5 shows that the intermediate (GC_Int_) and the tautomer product (G*C*) follow a similar trend, although the *E*_+*x*_ field further reduces the average relative energy of the intermediate by ∼2.5 kcal mol^−1^. The most apparent reason for the stabilisation of the intermediate, *i.e.*, it moving closer in energy to the initial reactant (GC), is because GC_Int_ has a similar configuration to the single proton transfer product zwitterion (G^−^C^+^), which has a polarity that aligns favourably within the *E*_+*x*_ field. Such an observation explains why single proton transfer reactions, which produce the G^−^C^+^ zwitterion, occur more frequently over the double proton transfer reaction in the *E*_+*x*_ field. The stability of the intermediate (GC_Int_) in the *E*_+*x*_ field is further demonstrated by a comparatively large forward Gibbs energy activation barrier for the second step (Δ*G*^‡^_b,f_ = 1.28 ± 1.01 kcal mol^−1^) and a positive average Gibbs energy reverse barrier for the first step (Δ*G*^‡^_a,r_ = 0.15 ± 0.78 kcal mol^−1^).

**Table 3:** The kinetic and thermodynamic properties of the stepwise double proton transfer in GC: the reaction energy Δ*G*, the respective forward and reverse barrier heights of the first step, Δ*G*^‡^_a,f_ and Δ*G*^‡^_a,r_, the second step, Δ*G*^‡^_b,f_ and Δ*G*^‡^_b,r_ (kcal mol^−1^), and the equilibrium constant (*K*). Mean values are calculated from the QM/MM ensemble using the B3LYP-XDM/aug-cc-pvdz/AMBER method at 300 K. The respective forward and reverse rate coefficients, *k*_f_ and *k*_r_ (s^−1^), and the half-life (*t*_1/2_) of the G*C* tautomer (s) are calculated using only the replicas from the QM/MM ensemble that consist of positive reverse Gibbs energy barriers (*E*_−*x*_, 2 out of 8 replicas; *E*_0_, 0 out of 21 replicas; and *E*_+*x*_, 2 out of 10 replicas). The applied field strengths are at 1.00 × 10^9^ V m^−1^. The standard deviation is denoted by *σ*

Stepwise DPT	Δ*G*^‡^_a,f_		Δ*G*^‡^_a,r_		Δ*G*^‡^_b,f_		Δ*G*^‡^_b,r_		Δ*G*		*K* × 10^−9^		*t*_1/2_ × 10^−13^		*k*_f_ × 10^5^		*k*_r_ × 10^12^	
	Mean	*σ*	Mean	*σ*	Mean	*σ*	Mean	*σ*	Mean	*σ*	Mean	*σ*	Mean	*σ*	Mean	*σ*	Mean	*σ*
*E*_−*x*_	11.61	1.59	−0.51	1.10	0.41	0.74	0.04	0.56	12.50	2.47	8.86	13.4	9.13	9.49	0.02	0.02	1.65	1.72
*E*_0_	10.38	1.09	−1.03	0.32	0.09	0.64	−0.87	0.58	12.37	1.28	5.55	9.21	—	—	—	—	—	—
*E*_+*x*_	9.91	1.57	0.15	0.78	1.28	1.01	−0.44	0.71	11.47	1.26	19.6	27.5	1.02	0.21	157	222	6.94	1.42

By contrast, the *E*_−*x*_ field stabilises the G*C* tautomer relative to the second transition state (GC^‡^_b_); we are unable to ascertain whether or not the relative Gibbs energy for the reverse barrier of the second step (Δ*G*^‡^_b,r_) is positive or negative (0.04 ± 0.56 kcal mol^−1^). Overall, the *E*_−*x*_ field increases the average forward and reverse Gibbs energy barrier heights for the first and second steps by *ca.* 1 kcal mol^−1^ and 0.5 kcal mol^−1^, respectively. Consequently, the equilibrium constant in the *E*_−*x*_ field (8.86 × 10^−9^) is smaller than in the *E*_+*x*_ field (1.96 × 10^−10^), but the half-life of the G*C* tautomer is almost one order of magnitude larger (9 × 10^−13^ s as opposed to 1 × 10^−13^ s, respectively). In the absence of the electric field none of the QM/MM replicas showed positive values for both reverse barriers, Δ*G*_a,r_ and Δ*G*_b,r_. For this reason, we can only assume that the *E*_0_ half-life of the G*C* tautomer is smaller than the picoseconds measured in the presence of the *E*_±*x*_ field. This suggests that in the field free case, the G*C* tautomer is more likely to revert towards the canonical Watson–Crick form.

Another trend suggests that the mean Gibbs reaction energy (Δ*G*) in the *E*_−*x*_ field is larger than *E*_0_, which is larger than *E*_+*x*_. It is worth mentioning that this trend is uncertain since the upper and lower bounds for the mean Gibbs reaction energy in the case of *E*_+*x*_ (11.47 ± 1.26 kcal mol^−1^) and *E*_0_ (12.37 ± 1.28 kcal mol^−1^) lie wholly within the error associated with *E*_−*x*_ (12.50 ± 2.47 kcal mol^−1^).

#### 3.4.2 Concerted double proton transfer

The kinetics and thermodynamics of the concerted proton transfer mechanism in GC in electric fields (*E*_+*x*_ and *E*_−*x*_) at 1.00 × 10^9^ V m^−1^ compared to the field-free instance are given in Table 4. The synchronous nature of the concerted DPT pathway is demonstrated in Fig. S6 of the ESI,† whereby the hydrogen bond lengths of the transition state indicate that both protons are transferred simultaneously. The concerted double proton transfer mechanism occurred most frequently in the *E*_−*x*_ field (17 times), three times in the *E*_0_ case, and only once in the *E*_+*x*_ field. Because of this, there are no reported errors for the values pertaining to *E*_+*x*_ field in Table 4 and Fig. 5, as well as large standard deviations reported for the field-free scenario (*E*_0_). Unfortunately, the errors with the associated mean forward energy barrier (Δ*G*^‡^_f_) in the *E*_−*x*_ field (12.79 ± 1.18 kcal mol^−1^) lie wholly within the large errors of the *E*_0_ case (12.46 ± 1.86 kcal mol^−1^); this is the same for the mean reverse energy barrier (Δ*G*^‡^_r_) at *E*_0_ (1.01 ± 1.45 kcal mol^−1^) and *E*_−*x*_ (0.38 ± 1.08 kcal mol^−1^). For this reason, it is not possible to confidently suggest any trends between the effects of the *E*_−*x*_ or *E*_+*x*_ fields on the thermodynamics and kinetics of the concerted double proton transfer mechanism. Nonetheless, the mean equilibrium constant (*K*) is one order of magnitude larger in the *E*_−*x*_ field than the *E*_0_ and *E*_+*x*_ fields. There are no clear correlations with respect to the G*C* tautomer half-life and the electric field polarity; the G*C* half-life is 5 ps at *E*_0_, 1 ps at *E*_−*x*_, and not evaluated in the *E*_+*x*_ field, due to the negative reverse Gibbs energy barrier (−0.86 kcal mol^−1^).

**Table 4:** The kinetic and thermodynamic properties of the concerted double proton transfer reaction in GC: the reaction energy, Δ*G*, the respective forward and reverse barrier heights, Δ*G*^‡^_f_ and Δ*G*^‡^_r_ (kcal mol^−1^), and the equilibrium constant, *K*. Mean values are calculated from the QM/MM-ensemble using the B3LYP-XDM/aug-cc-pvdz/AMBER method at 300 K. The respective forward and reverse rate coefficients *k*_f_ and *k*_r_ (s^−1^), and the half-life (*t*_1/2_) of the G*C* tautomer (s) are calculated using only the replicas from the QM/MM ensemble that have positive reverse Gibbs energy barriers (*E*_−*x*_, 7 out of 17 replicas; *E*_0_, 2 out of 3 replicas; and *E*_+*x*_, 0 out of 1 replica). The applied field strengths are at 1.00 × 10^9^ V m^−1^. The standard deviation is denoted by *σ* (where *σ* = ‘—’, the sample size consists of a single replica)

Concerted DPT	Δ*G*^‡^_f_		Δ*G*^‡^_r_		Δ*G*		*K* × 10^−9^		*t*_1/2_ × 10^−12^		*k*_f_ × 10^4^		*k*_r_ × 10^12^	
	Mean	*σ*	Mean	*σ*	Mean	*σ*	Mean	*σ*	Mean	*σ*	Mean	*σ*	Mean	*σ*
*E*_−*x*_	12.79	1.18	0.38	1.08	12.42	1.52	26.2	68.3	1.42	1.68	6.81	11.2	2.41	2.07
*E*_0_	12.46	1.86	1.01	1.45	11.44	0.61	7.99	8.09	4.57	6.29	0.53	0.71	2.75	3.78
*E*_+*x*_	11.33	—	−0.86	—	12.19	—	1.32	—	—	—	—	—	—	—

#### 3.4.3 Single proton transfer

The kinetics and thermodynamics for the GC single proton transfer reaction in the *E*_+*x*_ and *E*_−*x*_ fields at 1.00 × 10^9^ V m^−1^ compared to the field-free scenario are given in Table 5. The hydrogen bond lengths for the stationary points involved in the SPT pathway are given in Fig. S7 of the ESI† and show that the zwitterion product G^−^C^+^ has a similar structure to the stepwise DPT intermediate (GC)_Int_. There were no cases of single proton transfer in the *E*_−*x*_ field and only one case of single proton transfer in the absence of the electric fields. For this reason, there are no standard deviations to report for the field-free case (*E*_0_) nor any results to compare to in the *E*_−*x*_ field. It is worth mentioning that all of the thermodynamic and kinetic properties that were obtained for the *E*_0_ instance lie wholly within the standard deviation errors for the *E*_+*x*_ field. Similar to the concerted double proton transfer reaction, the lack of statistically robust data in the case of *E*_0_ indicates that it is not possible to confidently draw a comparison between the kinetics and thermodynamics of the single proton transfer in the presence and absence of the *E*_+*x*_ field. Nonetheless, the results in Table 5 suggest that the *E*_+*x*_ field increases the thermodynamic population of the G^−^C^+^ single proton transfer zwitterion when compared to *E*_0_.

**Table 5:** The kinetic and thermodynamic properties of the single proton transfer reaction in GC: The reaction energy, Δ*G*_rxn_, the respective forward and reverse barrier heights, Δ*G*^‡^_f_ and Δ*G*^‡^_r_ (kcal mol^−1^), and the equilibrium constant, *K*. Mean values are calculated from the QM/MM-ensemble using the B3LYP-XDM/aug-cc-pvdz/AMBER method at 300 K. The respective forward and reverse rate coefficients *k*_f_ and *k*_r_ (s^−1^) and the half-life (*t*_1/2_) of the G^−^C^+^ zwitterion (s) are calculated using only the replicas from the QM/MM ensemble that have positive reverse Gibbs energy barriers (*E*_0_, 0 out of 1 replica and *E*_+*x*_, 4 out of 14 replicas). The applied field strengths are at 1.00 × 10^9^ V m^−1^. The standard deviation is denoted by *σ* (where *σ* = ‘—’, the sample size consists of a single replica)

SPT	Δ*G*^‡^_f_		Δ*G*^‡^_r_		Δ*G*		*K* × 10^−6^		*t*_1/2_ × 10^−15^		*k*_f_ × 10^10^		*k*_r_ × 10^14^	
	Mean	*σ*	Mean	*σ*	Mean	*σ*	Mean	*σ*	Mean	*σ*	Mean	*σ*	Mean	*σ*
*E*_−*x*_	—	—	—	—	—	—	—	—	—	—	—	—	—	—
*E*_0_	7.56	—	−0.71	—	8.27	—	0.95	—	—	—	—	—	—	—
*E*_+*x*_	8.18	1.45	−0.14	0.42	8.32	1.64	25.3	73.6	6.97	8.26	8.23	14.1	4.05	4.06

The single proton transfer reaction has a lower forward Gibbs energy barrier than either of the aforementioned double proton transfer pathways. For this reason, the equilibrium population of the G^−^C^+^ zwitterion (∼10^−6^) is three orders of magnitude larger than the G*C* tautomer (∼10^−9^). Despite this, the average reverse Gibbs energy barrier for the single proton transfer process remains negative in the *E*_+*x*_ field.

### 3.5 Uncertainty quantification

**Fig. 7:**
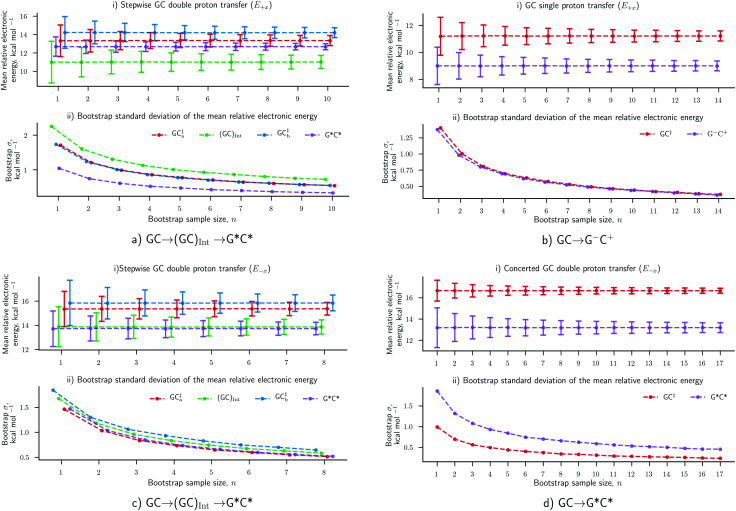
Bootstrapping analysis of QM/MM replica numbers (*n*) per relative electronic energies for (a) *E*_+*x*_ stepwise double proton transfer, (b) *E*_+*x*_ single proton transfer, (c) *E*_−*x*_ stepwise double proton transfer and (d) *E*_−*x*_ concerted double proton transfer reactions. (i) The mean bootstrap relative electronic energies, against the number of replicas used; the error bars are the bootstrap standard deviation. (ii) The bootstrap standard deviation, as shown in (a), against the number of replicas used. The *E*_+*x*_ and *E*_−*x*_ external electric fields are applied at 1.00× 10^9^ V m^−1^.

The results concerning the weak electric fields (10^4^ and 10^7^ V m^−1^), as well as the stronger ones (10^9^ V m^−1^) in the *y*- and *z*-direction, have been shown to have a negligible influence on the proton transfer energetics. For this reason, these results are considered to be as equally statistically robust as the results that were obtained in the absence of an external electric field (*E*_0_) in our previous work.^6^ However, the electric fields in the positive and negative *x*-direction at 1.00 × 10^9^ V m^−1^ have reported a vastly different ratio of proton transfer reactions when compared to the *E*_0_ scenario, therefore, their newly associated errors need to be evaluated. Here, we utilise the bootstrap statistic method to assess whether or not the same number of replicas (25) have been a suitable size for the QM/MM-ensemble to ensure the statistically relevant study of proton transfer in the presence of the *E*_±*x*_ field at 1.00 × 10^9^ V m^−1^. The bootstrap errors associated with mean relative electronic energies of the QM/MM geometry optimised transition states, intermediates (if applicable), and products, for the GC proton transfer reactions are calculated within the *E*_±*x*_ field at 1.00 × 10^9^ V m^−1^. The bootstrap analysis for the positive (*E*_+*x*_) field is given in Fig. 7a and b for the stepwise double proton transfer and the single proton transfer reactions, respectively. The bootstrap analysis for the negative (*E*_−*x*_) field is given in Fig. 7c and d for the stepwise double proton transfer and the concerted double proton transfer reactions, respectively.

In the absence of the electric fields, the stepwise GC double proton transfer mechanism was by far the most frequently occurring in the QM/MM-ensemble (21 replicas). Therefore, due to a large number of stepwise replicas obtained, that mechanism was sampled effectively and consequently, the bootstrap standard deviation errors were very small (*ca.* 0.25 kcal mol^−1^).^6^ By contrast, the *E*_±*x*_ electric fields further divide each QM/MM-ensemble into different proton transfer mechanism ‘subsets’ that are comprised of 12 replicas on average (as opposed to 21). Fewer replicas per proton transfer mechanism constitute a less effectively sampled system and are therefore expected to produce larger errors. An example of this is shown in the stepwise double proton transfer mechanisms within the *E*_+*x*_ (Fig. 7a) and *E*_−*x*_ field (Fig. 7c); these two subsets are the smallest, comprised of a respective 10 and 8 replicas, and provide for the largest bootstrap standard deviation errors of ∼0.75 kcal mol^−1^. Overall, the bootstrap standard deviation errors in the presence of the *E*_±*x*_ fields are some two to three times larger than the *E*_0_ scenario yet remain within a 1 kcal mol^−1^ threshold.

## 4 Discussion

### 4.1 Comparison with previous literature

The multiscale QM/MM model utilised in this work provides an accurate description of a realistic DNA base pair and makes approximations that are a substantial improvement on previous QM gas-phase calculations. In doing so, the surrounding solvent and the phosphate backbone for a DNA dodecamer strand are explicitly modelled using the well parametrised AMBER force field. We have shown that the *E*_+*x*_ field at 1.00 × 10^9^ V m^−1^ reduces the relative energy of the proton transfer transition state(s) by approximately 1 kcal mol^−1^ when compared to *E*_0_. By contrast, the DPT in an isolated gas phase GC base-pair modelled by Cerón-Carrasco *et al.*^10^ showed a much larger decrease in transition state energy from *ca.* 14.5 kcal mol^−1^ (in *E*_0_) to *ca.* 7.0 kcal mol^−1^ (in the *E*_+*x*_ direction at 1.03 × 10^9^ V m^−1^) when using the M06-2X and MP2/6-311++G(d,p) QM methods. Cerón-Carrasco *et al.*^11^ did not reproduce this large deviation (*ca.* 7 kcal mol^−1^) in relative transition state energy when they modelled a gas phase GC base pair embedded in a DNA double-helix trimer using the semi-empirical QM/MM (M06-2X/6-311++G(d,p):M06-2X/6-31G(d):PM6) method. This more realistic embedded base pair model, although still lacking an explicit solvent, now showed that the *E*_+*x*_ 1.03× 10^9^ V m^−1^ field has a negligible influence on the relative transition state energy compared to *E*_0_.

Arabi and Matta^12^ modelled the DPT in an isolated gas-phase GC base pair using B3LYP/6-311++G** and also showed that the relative transition state energy changes by a negligible amount in the presence of the *E*_+*x*_ 1.29 × 10^9^ V m^−1^ electric field. We have calculated Gibbs energy barriers within 1–2 kcal mol^−1^ to those calculated by Arabi and Matta,^12^ who had also used the B3LYP functional as their QM method. By comparison, the other studies^10,11^ that had opted for the M06-2X functional have estimated reaction barriers larger than ours by *ca.* 4 kcal mol^−1^. This implies that choosing the QM method is a key factor in determining the size of the proton transfer activation energy barriers. Previous studies^10–12^ and this work have shown that the Gibbs reaction energy, *i.e.*, the energy of G*C* relative to canonical GC, decreases by 0.1 to 0.9 kcal mol^−1^ in the presence of the *E*_+*x*_ field at *ca.* 1 × 10^9^ V m^−1^. In addition, the Gibbs energy of G*C* relative to GC has consistently been calculated to between 9 to 12 kcal mol^−1^,^10–12^ irrespective of the chosen QM method. However, a single gas phase QM model cannot provide a reasonable estimation of the errors associated with the barrier height calculations. In accordance with our previous work,^6^ the ensemble-based QM/MM methodology applied here demonstrates that multiple proton transfer reactions occur in GC in the presence of different electric fields. Such an observation is unobtainable from previous studies in the literature, none of which have quantified the uncertainty in their simulations. By contrast to the previous studies, we found the concerted DPT mechanism to be a subsidiary reaction in the *E*_+*x*_ field (only one replica exhibits this process). This work shows that the equilibrium constant for the stepwise GC → G*C* DPT tautomerism increases by an order of magnitude within the *E*_+*x*_ field at 1.00 × 10^9^ V m^−1^ and is consistent with previous studies.^11,12^

This work has also shown that electric fields of 1.00 × 10^9^ V m^−1^ (*E*_+*x*_) are large enough to promote the formation of the single proton transfer G^−^C^+^ zwitterion instead of the double proton transfer G*C* tautomer. By contrast, Cerón-Carrasco and Jacquemin^11^ calculated 4.11 × 10^9^ V m^−1^ to be the turning point for the preferential formation of the G^−^C^+^ zwitterion. We report zero cases of the G^+^C^−^ zwitterion occurring, irrespective of exposure to the 1.00 × 10^9^ V m^−1^ (*E*_−*x*_) electric field. This observation is aligned with that of Cerón-Carrasco and Jacquemin,^11^ who found the G^+^C^−^ zwitterion to form at a larger electric field strength (3.09 × 10^9^ V m^−1^) than the ones studied here. This hypothesis can only be confirmed if the ensemble-based multiscale model is adapted to include a larger range of electric fields.

### 4.2 Biological implications

As demonstrated by the molecular dynamics section of this study, electric fields (≤1.00 × 10^9^ V m^−1^) in 10 ns pulses have a negligible effect on the structural properties of DNA. Such short timescales have practical medical applications, whereby electrical pulses are applied to cells in the nanosecond duration, the shortest of which are applied for 1–10 ns at strengths of 10^6^–10^7^ V m^−1^.^23,25,26^ Nanosecond electric pulses are presumed to affect the morphology of nuclei and may lead to fragmentation in DNA, the extent of which is poorly defined.^26^ There is no evidence to suggest that pulse duration is directly linked to the effects nanosecond electric fields have on structural properties of DNA. With that in mind, recent experimental evidence, supported by MD simulation, has shown that 10 ns pulses increase the yield of *in vivo* gene delivery in a nontoxic way.^64^ The simulations in this paper do not consider the effects of a surrounding cell membrane but do conclude that short nanosecond pulses (provided they are ≤10^9^ V m^−1^) will not break down or fragment aqueous DNA in ambient conditions.

The QM/MM part of this study shows that weaker external electric fields (≤1.00 × 10^7^ V m^−1^) have no effect on the proton transfer mechanisms within the GC base pair. We, therefore, demonstrate that the electric fields most commonly applied in medical practices will neither increase the concentration nor decrease the lifetimes of the mutagenic G*C* tautomer. Therefore, the exposure to electric fields less than or equal to 10^7^ V m^−1^ is unlikely to be a contributing factor towards the onset of genetic diseases. A similar conclusion is drawn for the electric fields at 1.00 × 10^9^ V m^−1^. It is when the electric field is oriented parallel to the base pair hydrogen bond axis (labelled as the *x*-direction) that the proton transfer reactions are affected the most. Even then, the G*C* tautomer is shown to have a maximum half-life in the picosecond range, and an even smaller half-life in the femtosecond range for the G^−^C^+^ zwitterion. With this in mind, we predict that short pulses of external electric fields up to 1.00 × 10^9^ V m^−1^ over a duration of 1 to 10 ns can be applied in medical practices without increasing the probabilities of point mutations in DNA *via* the Löwdin mechanism.

The trends observed in this paper suggest that the transient tautomer products may be further stabilised by more intense electric fields than the maximum strength studied (>1.00 × 10^9^ V m^−1^). Earlier QM studies^11,12^ indicate that external electric fields of strength ∼5 × 10^9^ V m^−1^ are large enough to stabilise the proton transfer products relative to the canonical Watson–Crick base pairs. Nonetheless, electric fields of such a large magnitude (>1.00 × 10^9^ V m^−1^) are unlikely to occur naturally *in vivo*^27,28,65,66^ and would provide a substantial challenge to practically apply at the desired orientation.^16^ Compounding this, previous classical MD simulations^62^ under ambient conditions have shown that the application of electric fields stronger than 3.09 × 10^9^ V m^−1^ completely disrupt and permanently unwind the DNA double helix when applied in 10 ps or longer pulses.

## 5 Conclusion

We have demonstrated that weak electric field strengths (≤10^7^ V m^−1^) do not affect the stability of the GC proton transfer resonance forms. Typical medical applications rarely exceed field strengths of 10^7^ V m^−1^ and therefore have virtually no chance to induce errors in DNA replication *via* the Löwdin mutation mechanism. On the other hand, oriented external electric fields (provided they are 1.00 × 10^9^ V m^−1^) are shown to increase the likelihood of certain resonance forms occurring: *E*_+*x*_ promotes the formation of the G^−^C^+^ zwitterion, while *E*_−*x*_ promotes the formation of the G*C* tautomer. Although certain proton transfer reactions are more likely to occur than others in different electric field orientations, the products themselves (G^−^C^+^ and G*C*) are still calculated to be transient species with lifetimes less than several picoseconds. In the presence of the electric fields studied in this work, the proton transfer products are unlikely to contribute towards mutations as their lifetimes are approximately three to five orders of magnitude smaller than the nanosecond timescale it takes for DNA to open during replication.^7^

The upper bound estimate of the electric field strength within the phospholipid bilayer of a cell is within the range of 10^8^–10^9^ V m^−1^.^27,28^ From an abiogenetic perspective, this would suggest that the very large electric fields generated within biological membranes are just short of stabilising the otherwise transient mutagenic tautomers. The process of gene therapy *via* electroporation involves the passing of DNA through the phospholipid bilayer of a cell and is often catalysed by a nanosecond pulsed external electric field (∼10^7^ V m^−1^). This work demonstrates that the proton transfer tautomers within DNA that are exposed to electric fields ≤1.00 × 10^9^ V m^−1^ will remain equally metastable when compared to the no-field scenario.

## Conflicts of interest

There are no conflicts to declare.

## Supplementary Material

CP-023-D0CP06218A-s001
