# Supplementary material for: The influence of external electric fields on proton transfer tautomerism in the guanine–cytosine base pair
Source: Phys Chem Chem Phys. 2021 Feb 23;23(10):6252–65. doi: 10.1039/d0cp06218a (PMC8330266; doi:10.1039/d0cp06218a)
Supplement: CP-023-D0CP06218A-s001 [file CP-023-D0CP06218A-s001.pdf]

Electronic Supplementary Material (ESI) for Physical Chemistry Chemical Physics.
This journal is © the Owner Societies 2021

Supplementary Material: The influence of external electric fields on proton transfer tautomerism in the guanine-cytosine base pair

Gheorghiu A.[1], Coveney P. V.[1,2]*, Arabi A. A.[1,3]*

[1]*Centre for Computational Science, University College London, United Kingdom*
[2]*Informatics Institute, University of Amsterdam, Netherlands*
[3]*College of Medicine and Health Sciences, United Arab Emirates University, United Arab Emirates*

February 23, 2021

Introduction

This document provides further data and information in support of the main article. The first section extends the all-atom molecular dynamics study and investigates how the external electric fields affect the hydrogen bond lengths in GC. The second section details how the strength of the external electric fields applied in the QM/MM methodology was verified. The third section reports the ensemble of GC proton transfer QM/MM reaction coordinates in the presence of weak electric fields (10^4 to 10^7 V/m). The fourth and fifth sections describe the ensemble of GC proton transfer QM/MM reaction coordinates in the presence of strong electric fields in the $\pm y$ and $\pm z$ directions, respectively. The sixth and seventh sections display how the GC hydrogen bond lengths change during the concerted double and single proton transfer reactions.

1 Investigating the external electric field Homogeneity

To calculate the electric field strength produced by the charged plates in the QM/MM simulation cell, the force was calculated on a single sodium ion at the system's origin. The forces on the sodium ion provided for a quantified description of the external electric field at that position. Table S1 shows that at the origin, the electric fields obtained from the point charge plate model are very close to the intended range of electric field strengths. At fields strengths above 10^6 V/m, the point-charge model is within two decimal places of the intended electric field strengths.

Table S1: The charge of each dummy atom in the electric field plates, the intended electric field strength, and the actual electric field strength generated by the point-charge field model at the simulation cell origin.

Dummy atom charge (a.u.)	Intended field strength (V/m)	Obtained field strength (V/m)
1.83×10^{-6}	1.00×10^{4}	1.09×10^{4}
1.83×10^{-5}	1.00×10^{5}	9.85×10^{4}
1.83×10^{-4}	1.00×10^{6}	1.00×10^{6}
1.83×10^{-3}	1.00×10^{7}	1.00×10^{7}
1.83×10^{-2}	1.00×10^{8}	1.00×10^{8}
1.83×10^{-1}	1.00×10^{9}	1.00×10^{9}

Since the electric field is generated by point charges, it is clear that the electric field between the two plates is not perfectly homogeneous. An inhomogeneous electric field does not detract from the validity of this methodology since electric fields found in real life are never perfectly homogeneous. The homogeneity of the external electric fields generated by the charged plates are quantified by calculating the forces on a sodium ion at different spatial coordinate intervals within the simulation cell. The results (displayed in Figure S1) show that the E_{+x} electric field varies more in the axis parallel to the field direction (x) than in the directions perpendicular to the field (y and z). This is especially pronounced at positions in the cell more than 15 Å from the origin. Such an increase in the electric field strengths is expected since moving in the x-axis brings the ion closer to the charged plates. The perturbation in the electric field strength is less than 1% at positions that are ±5 Å from the origin; such a distance is large enough to fit an entire GC base pair (∼10

Å) comfortably. Identical homogeneity patterns to those observed in E_{+x} are also obtained for the other external electric field directions (E_{-x}, E_{+y}, E_{-y}, E_{+z} and E_{-z}).

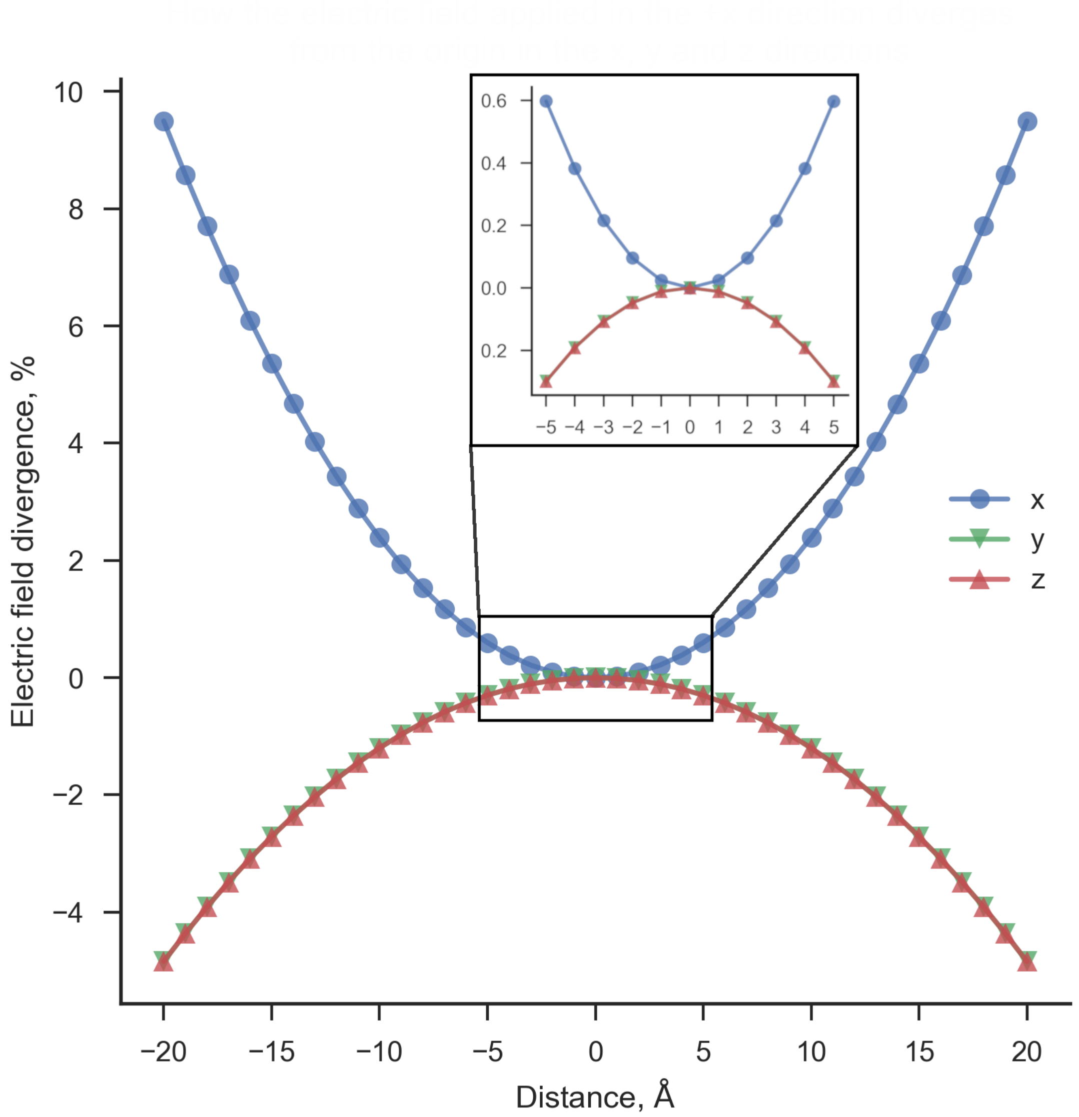

Figure S1: The variation of the positive x-direction electric field strength (E_{+x}) at different positions in the simulation cell in the principle x-, y- and z-axis.

2 GC Hydrogen Bond Lengths in the Presence of External Electric FIelds

The average bond lengths for each GC base pair hydrogen bond in the absence and presence of electric fields (in the E_{+x} direction) are shown in Figure S2. When comparing the no field scenario (0 V/m) to the largest external electric field strength (10^9 V/m), the average GC hydrogen bond lengths change by less than 0.015 Å. Essentially, the mean hydrogen bond lengths at 10^9 V/m are within the reported standard deviation errors in the absence of the field.

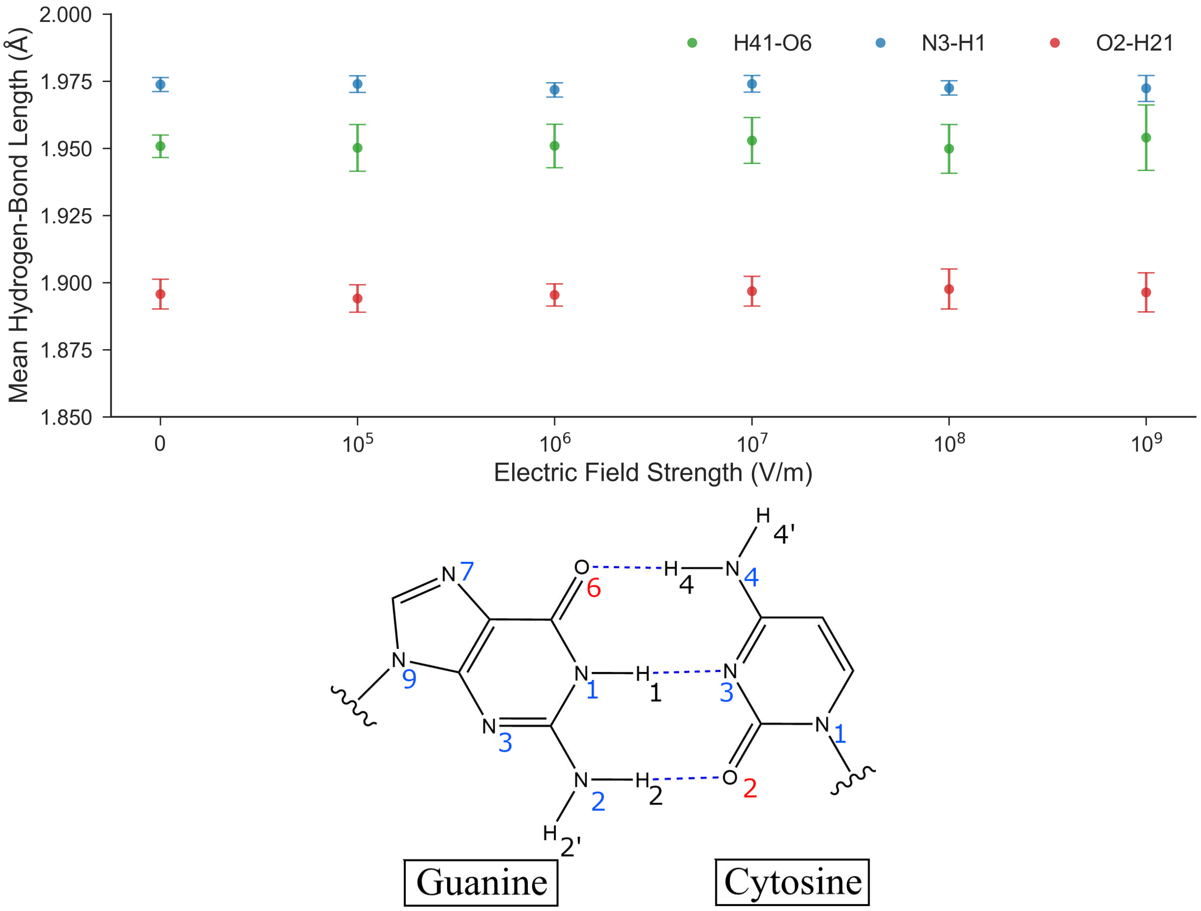

Figure S2: The mean GC hydrogen bond lengths (Å) in different external electric field strengths (V/m). The fields are applied in the positive x-direction (E_{+x}). Each point is the mean hydrogen-bond length of ten, 10 ns, all-atom MD replicas; the error bars represent the standard deviation. The mean hydrogen bonds experience a negligible change to their lengths in the presence of the external electric field.

3 Weak Electric Fields in the x-direction: Proton Transfer Reaction Coordinates

The 25 individual QM/MM climbing image nudged elastic band (CI-NEB) pathways for the proton transfer reactions in GC in the E_{+x} external electric field at $1.00{\times}10^4$ V/m and $1.00{\times}10^7$ V/m, as well as the instance of no field (E_0), are shown in Figure S3. Every QM/MM replica shows that the $1.00{\times}10^4$ V/m has a negligible impact on the energetics of proton transfer in the GC base pair. A similar trend is observed for the $1.00{\times}10^7$ V/m electric field, with the exception of one replica (labelled no. 113 in Figure S3), whereby the double proton transfer activation energy and the reaction energy are lowered by ca. 3 kcal/mol and ca. 8 kcal/mol, respectively. Replica no. 113 is considered to be an anomalous result, as the change in energetics were far greater than all the other 24 replicas in the ensemble. Therefore, it is concluded that electric fields with strengths between $1.00{\times}10^4$ to $1.00{\times}10^7$ V/m have a negligible effect on the proton transfer reaction in GC.

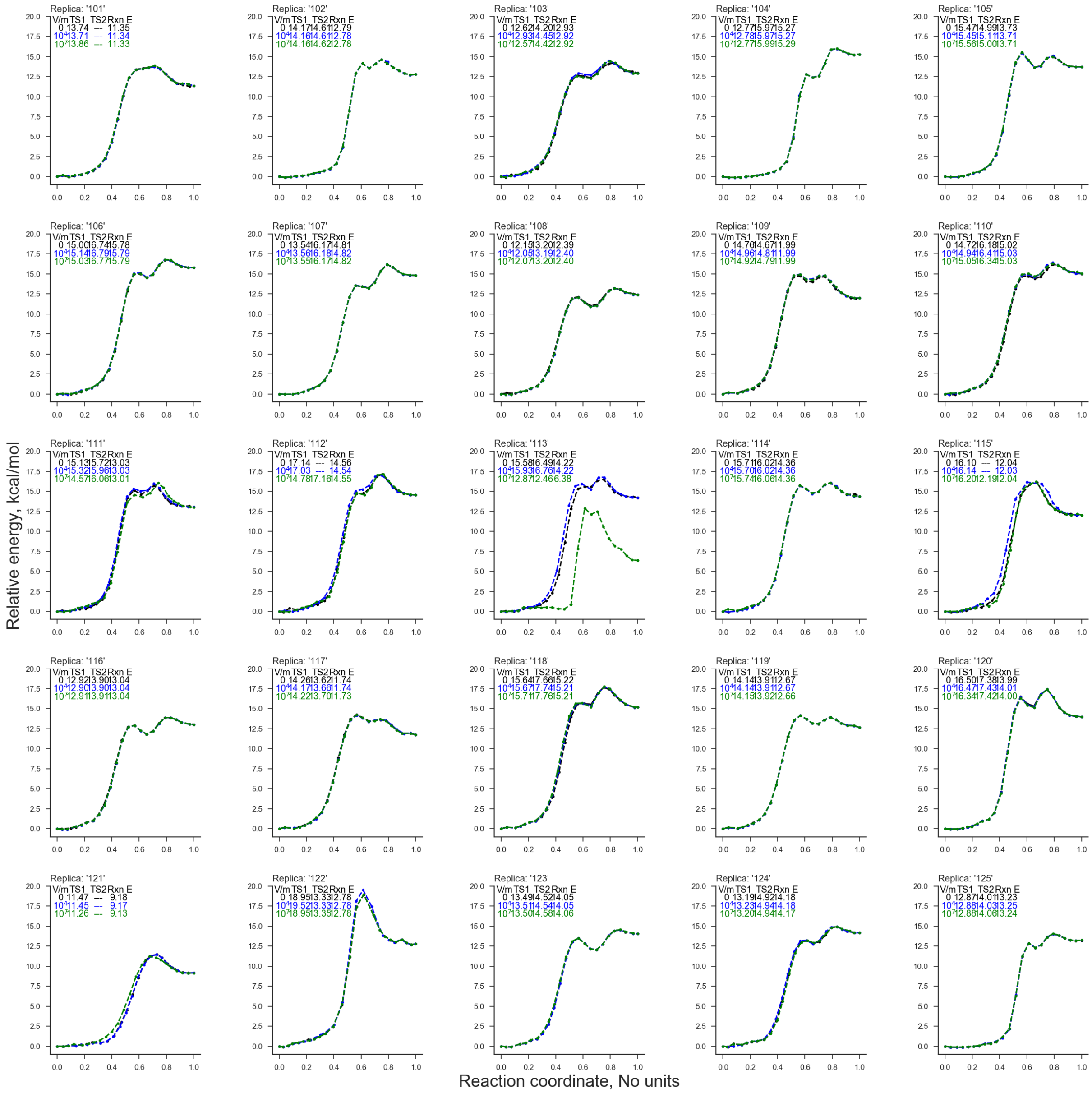

Figure S3: The 25 QM/MM CI-NEB reaction coordinates for the proton transfer in the GC base pair: in the absence of external electric fields (black), in the presence of an external electric field applied in the positive x-direction (E_{+x}) at strength 10^4 V/m (blue) and 10^7 V/m (green). The reaction coordinate is normalised (no units) and the electronic energy relative to the reactant (GC) is given in kcal/mol. Every replica, except no. 113, experiences a negligible change in energetics in the presence of 10^4 V/m and 10^7 V/m OEEFs when compared to no field.

4 External Electric Fields in the y-direction: Proton Transfer Reaction Coordinates

This section investigates how the electric fields in the y-direction at 10^9 V/m influence the energetics of the proton transfer reactions in GC. The orientation of the electric field is orthogonal to the base pair hydrogen bonds and parallel to the DNA dodecamer helix.

The 25 individual QM/MM CI-NEB pathways for the proton transfer reactions in GC at 1.00×10^9 V/m in the presence the E_{+y} and E_{-y} fields, as well as the instance of no field (E_0), are given in Figure S4. On the whole, it is evident that the proton transfer reaction coordinates in both the positive and negative y-direction weakly perturb the energetics of the GC proton transfer when compared to the no field scenario. Two exceptions to this are the E_{+y} replicas numbered '104' and '115', which display a comparatively drastic change in the reaction coordinate profile. This is due to the inability for the optimisation algorithm to reach the appropriate level of convergence. Since this was only the case for two replicas out of a total 50 (E_{+y} and E_{-y} combined), the statistical significance of the aforementioned outliers is presumed to be small.

There are no clear distinctions, or trends, between the E_{-y} and E_{+y} fields and their effects on the relative transition state energies. Aside from several outliers, the y-direction electric fields do not modify the transition state energies by more than ±0.5 kcal/mol. For this reason, the transition state estimates were not subjected to further geometry optimisation, since the thermodynamics and kinetics of the proton transfer process in both the E_{+y} and E_{-y} fields are presumed to be similar to the instance of no field (E_0).

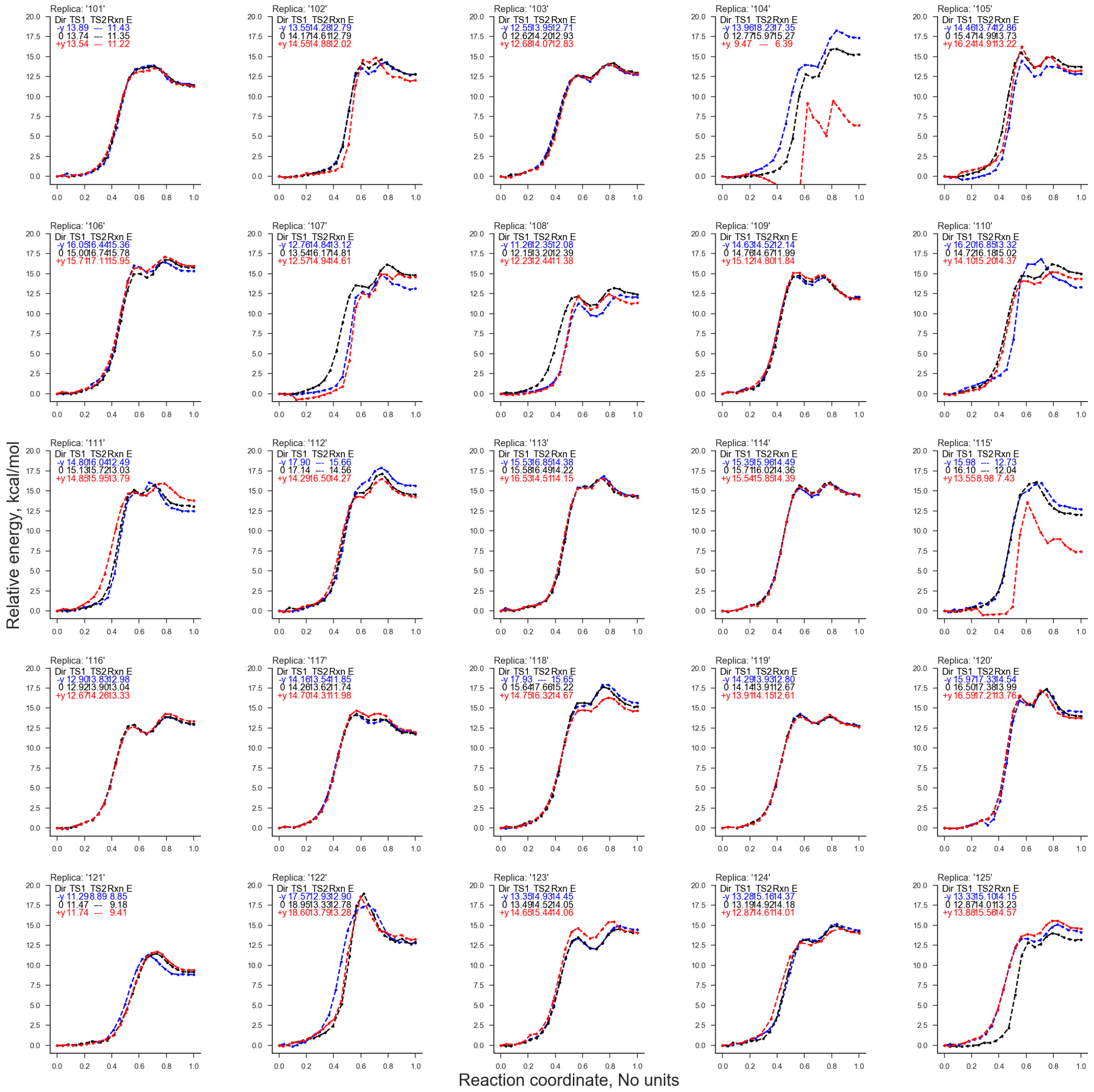

Figure S4: The 25 QM/MM CI-NEB reaction coordinates for the GC proton transfer reactions: in the absence of an external electric field (black) and the presence of an external electric field (10^9 V/m) in the E_{+y} (red) and E_{-y} (blue) directions. The reaction coordinate is normalised (no units) and the electronic energy relative to the reactant (GC) is given in kcal/mol. The majority of replicas show that the proton transfer energetics are either unaffected or at best weakly perturbed (by <0.5 kcal/mol), in the presence of the E_{+y} and E_{-y} electric field.

5 External Electric Fields in the z-direction: Proton Transfer Reaction Coordinates

This section considers the influence electric fields in the z-direction at 10^9 V/m have on the energetics of the proton transfer reactions in GC. The orientation of the z-direction field is on the same geometric plane as the GC base pair and is orthogonal to the hydrogen bonds.

The 25 individual QM/MM CI-NEB pathways for the proton transfer reactions in GC at 10^9 V/m in the presence of the E_{+z} and E_{-z} fields, as well as the instance of no field (E_0), are given in Figure S5. Approximately 15 of the QM/MM replicas show a negligible difference between the E_{+z} and E_{-z} fields when compared to the no field reaction coordinates.

Similar to the y-direction, there are no clear trends between how the E_{+z} and E_{-z} fields influence the relative transition state energies. For example, two replicas show that the E_{+z} field (Figure S5: red) increases the relative transition state energy by ca. 1.5 kcal/mol, while another three replicas show that the relative transition state energy is decreased by ca. 1.5 kcal/mol. These results suggest that the OEEFs in the z-direction have the potential to affect the kinetics and thermodynamics of the proton transfer reaction, but only at larger electric field strengths than 10^9 V/m. For now, the transition state estimates were not subjected to further geometry optimisation, since the thermodynamics and kinetics of the proton transfer process in both the E_{+z} and E_{-z} fields are presumed to be similar to the instance of no field (E_0).

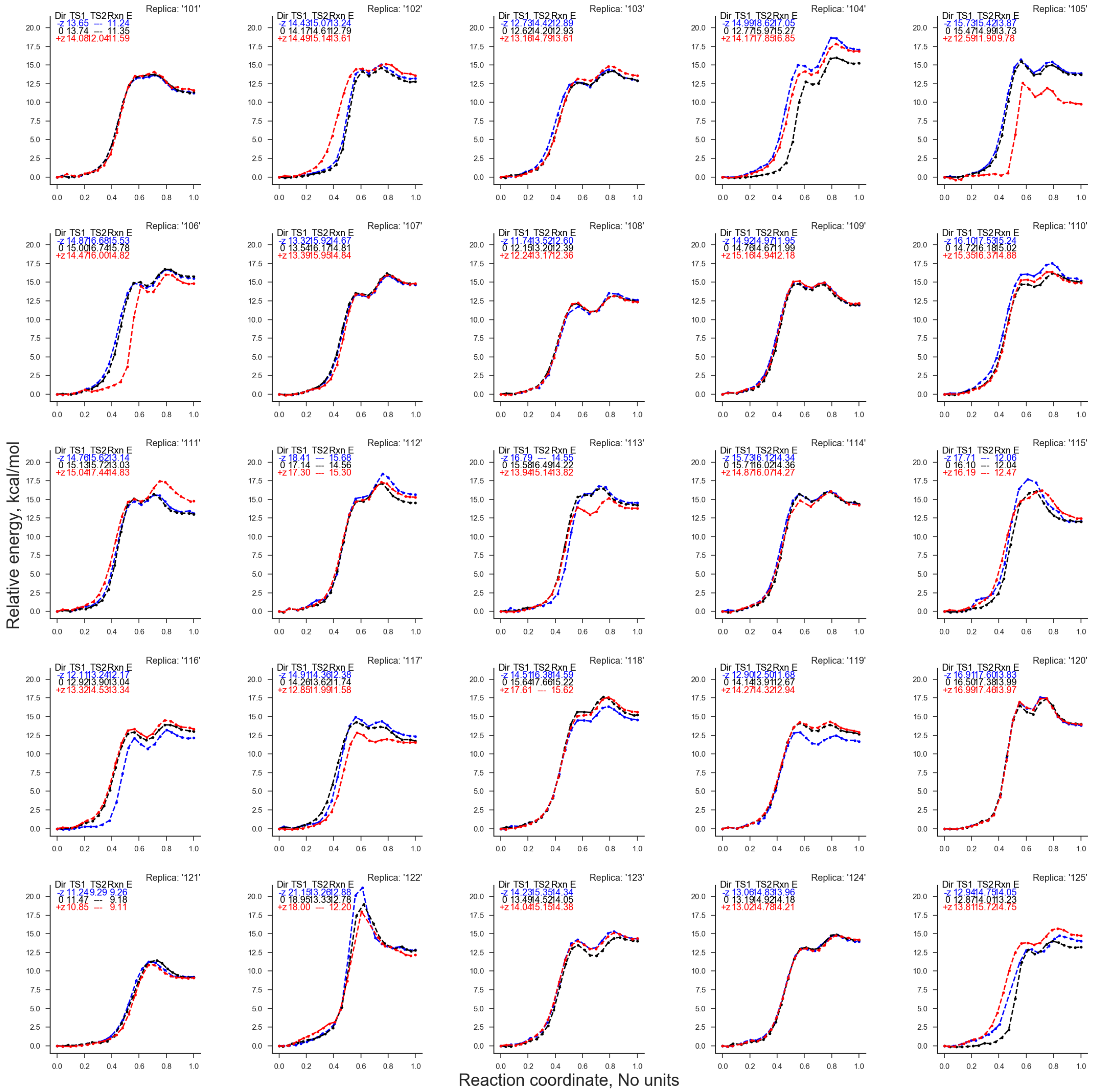

Figure S5: The 25 QM/MM CI-NEB reaction coordinates for the GC proton transfer reactions: in the absence of external electric fields (black) and the presence of an external electric field (10^9 V/m) in the E_{+z} (red) and E_{-z} (blue) directions. The reaction coordinate is normalised (no units) and the electronic energy relative to the reactant (GC) is given in kcal/mol. More than half of the replicas remain unchanged in the presence of the electric fields. Several replicas show a small perturbation to the electronic energy in the presence of the electric fields (although these rarely exceed 1.5 kcal/mol). There are no correlations between the effects of the E_{+z} and E_{-z} fields.

6 Concerted Double Proton Transfer Bond Lengths

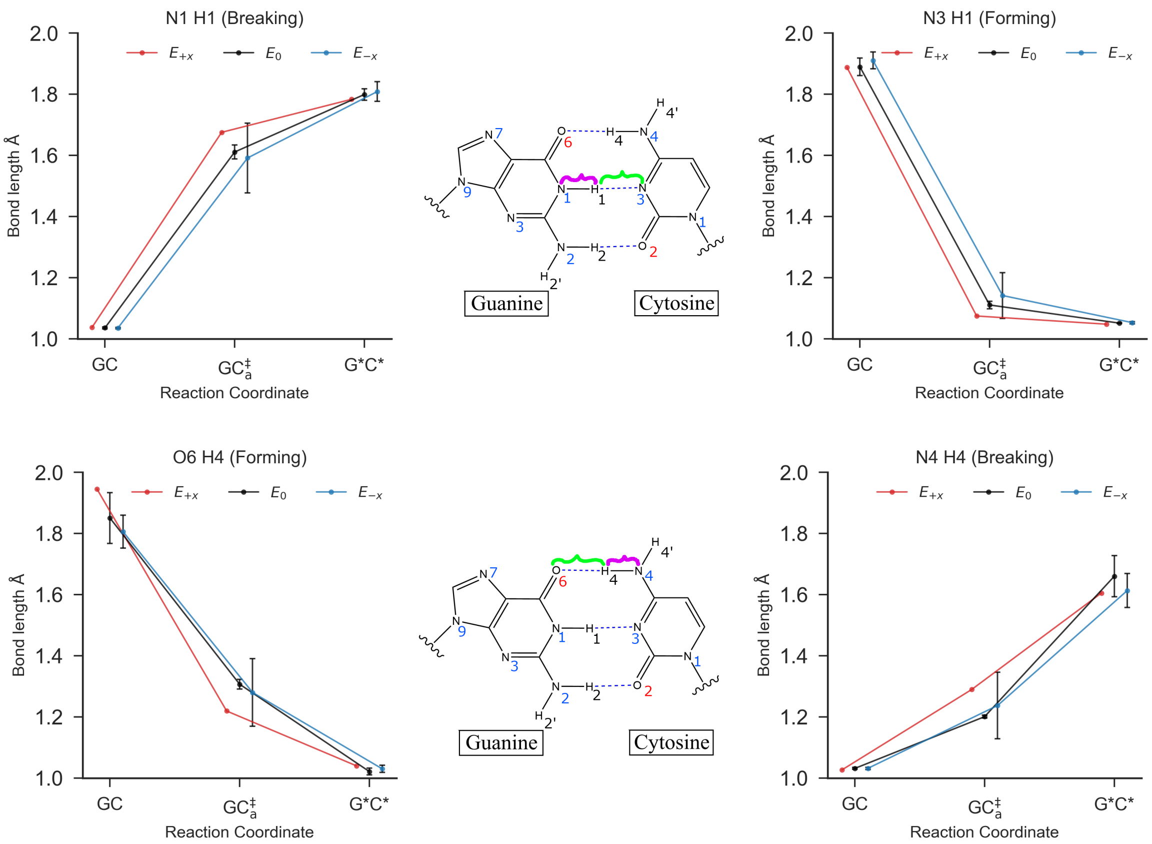

Figure S6: The mean hydrogen bond lengths for each stationary point in the concerted double proton transfer reaction in GC. The geometries for each stationary point are optimised using B3LYP+XDM/aug-cc-pvdz/AMBER. The bonds being broken (N4-H4 and N1-H1) are highlighted in purple, and the bonds that are formed (O6-H4 and N3-H1) are highlighted in green. The external electric fields (E_{+x} in red and E_{-x} in blue) are applied at $1.00{\times}10^9$ V/m, and no field (E_0) is given in black. The error bars are the standard deviation of the mean bond lengths.

The synchronous nature of the concerted DPT pathway is displayed in Figure S6, whereby the reaction proceeds via a transition state in which the inner H1 proton is transferred, and the outer H4 proton is simultaneously partially transferred. It is also shown that the hydrogen bond lengths for the G*C* formed via the concerted DPT mechanism are within the range of those formed by the stepwise DPT mechanism (Figure 6, main article).

7 Single Proton Transfer Bond Lengths

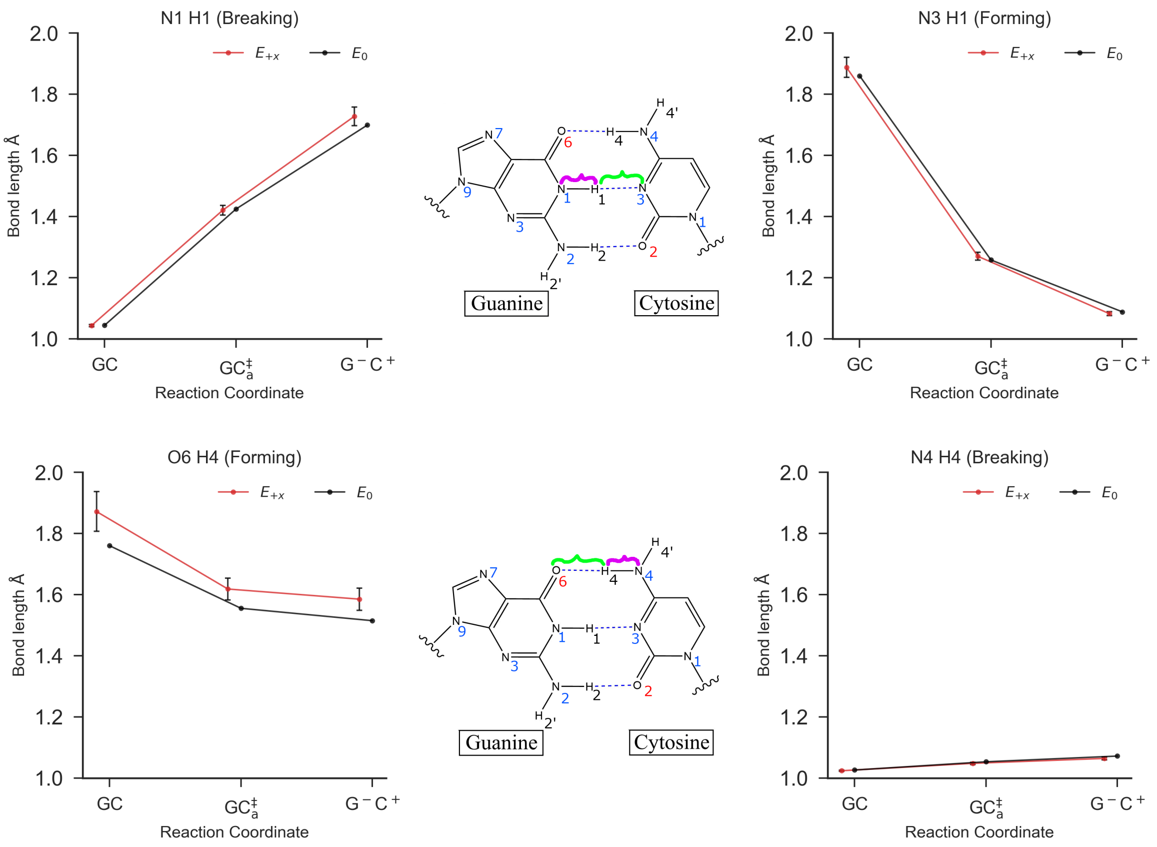

Figure S7: The mean hydrogen bond lengths for each stationary point in the single proton transfer reaction in GC. The geometries for each stationary point are optimised using B3LYP+XDM/aug-cc-pvdz/AMBER. The bonds being broken (N4-H4 and N1-H1) are highlighted in purple, and the bonds that are formed (O6-H4 and N3-H1) are highlighted in green. The E_{+x} external electric field (red) is applied at 1.00×10^9 V/m, and no field (E_0) is given in black (There was no single proton transfer mechanisms observed in the E_{-x} field and only one case in the absence of the field). The error bars are the standard deviation of the mean bond lengths.

Figure S7 shows that the single proton transfer reaction proceeds via the proton transfer of the inner H1 atom. The crucial difference, compared to DPT mechanisms, is that the H4 proton is not transferred from the cytosine to the guanine. Figure S7 also demonstrates the similarities between the geometries of the SPT zwitterion product, G^-C^+, and the stepwise DPT intermediate, $(GC)_{\mathrm{Int}}$ (as given in Figure 6, main article).
